# KBTBD11, encoding a novel PPARγ target gene, is involved in NFATc1 proteolysis by interacting with HSC70 and HSP60

**DOI:** 10.1038/s41598-022-24929-5

**Published:** 2022-11-24

**Authors:** Kazuhisa Watanabe, Ayumi Matsumoto, Hidetoshi Tsuda, Sadahiko Iwamoto

**Affiliations:** Division of Human Genetics, Center for Molecular Medicine, Jichi Medical University, 3311-1 Yakushiji, Shimotsuke, Tochigi 329-0498 Japan

**Keywords:** Biochemistry, Molecular biology, Molecular medicine

## Abstract

We previously revealed that *Kbtbd11* mRNA levels increase during 3T3-L1 differentiation and *Kbtbd11* knockdown suppresses whereas its overexpression promotes adipogenesis. However, how *Kbtbd11* mRNA is regulated during adipocyte differentiation and how the KBTBD11 protein functions in adipocytes remain elusive. This study aimed to examine the transcriptional regulatory mechanism of *Kbtbd11* during adipocyte differentiation, KBTBD11-interacting protein functions, and elucidate the role of KBTBD11 in adipocytes. First, we identified the PPRE consensus sequences in the *Kbtbd11* exon 1- and intron 1-containing region and demonstrated that PPARγ acts on this region to regulate *Kbtbd11* expression. Next, we purified the KBTBD11 protein complex from 3T3-L1 adipocytes and identified heat shock proteins HSC70 and HSP60 as novel KBTBD11-interacting proteins. HSC70 and HSP60 inhibition increased KBTBD11 protein levels that promoted NFATc1 ubiquitination. These data suggest that HSC70 and HSP60 are involved in KBTBD11 stabilization and are responsible for NFATc1 regulation on the protein level. In summary, this study describes first the protein regulatory mechanism of NFATc1 through the HSC70/HSP60-KBTBD11 interaction that could provide a potential new target for the differentiation and proliferation of various cells, including adipocytes and tumors.

## Introduction

Nutritional refeeding reportedly increases *Kbtbd11* expression in the epididymal white adipose tissue (eWAT) and *Kbtbd11* expression is significantly increased in the eWAT of mice fed on a high-fat diet compared to normal those on a chow diet^1^. In addition, *Kbtbd11* expression is increased during adipocyte differentiation and the adenovirus-mediated *Kbtbd11* knockdown suppresses 3T3-L1 adipocyte differentiation, while *Kbtbd11* overexpression promotes it^1^. These results demonstrate that *Kbtbd11* exhibits an important role in mitotic clonal expansion (MCE), the early stage of 3T3-L1 adipocyte differentiation and that it influences adipocyte differentiation^1^. Furthermore, *Kbtbd11* expression is reportedly regulated by USF1, a lipid metabolism-related transcription factor^2^. Taken together, *Kbtbd11* expression levels seem to play an important role in MCE, influence triglyceride accumulation, and adipocyte differentiation.

KBTBD11 is a member of the BTB protein superfamily with an N-terminal BTB/POZ domain and a C-terminal Kelch-repeat domain. The BTB domain is responsible for protein–protein interaction for dimer formation and self-association, and it is involved in other protein–protein interactions^1^. The BTB domain mainly acts as a specific adapter for Cullin3, a RING-finger type E3 ubiquitin ligase that mediates ubiquitin-dependent proteasome-mediated degradation^3^. The Kelch-repeat forms the β-propeller, responsible for the formation of protein–protein interactions^4^. Based on the aforementioned pieces of evidence, KBTBD11 appears to be involved in E3 ubiquitin ligase-mediated events.

Although in our work, we focused on adipocyte differentiation, apart from adipocytes, *KBTBD11* mRNA and protein levels are reportedly increased during RANKL-induced osteoclast differentiation in RAW-D cells^5^. KBTBD11 is also known to be involved in osteoclastogenesis by regulating the nuclear factor of activated T cells 1 (NFATc1) protein ubiquitination via E3 ubiquitin ligase^5^. Therefore, KBTBD11 might be involved in cell differentiation by regulating NFATc1 via the E3 ubiquitin ligase.

However, it remains unclear how *Kbtbd11* gene expression is regulated during adipocyte differentiation and how KBTBD11 functions in adipocytes. In this study, we described a novel transcription factor for targeting *Kbtbd11* and identified KBTBD11-interacting proteins along with the role of their KBTBD11 complexes. This finding suggested that the regulation of KBTBD11 plays an important role in the differentiation of not only adipocytes but also other cells.

## Results

### Peroxisome proliferator-activated receptor gamma (PPARγ) upregulates *Kbtbd11* mRNA expression

**Figure 1:**
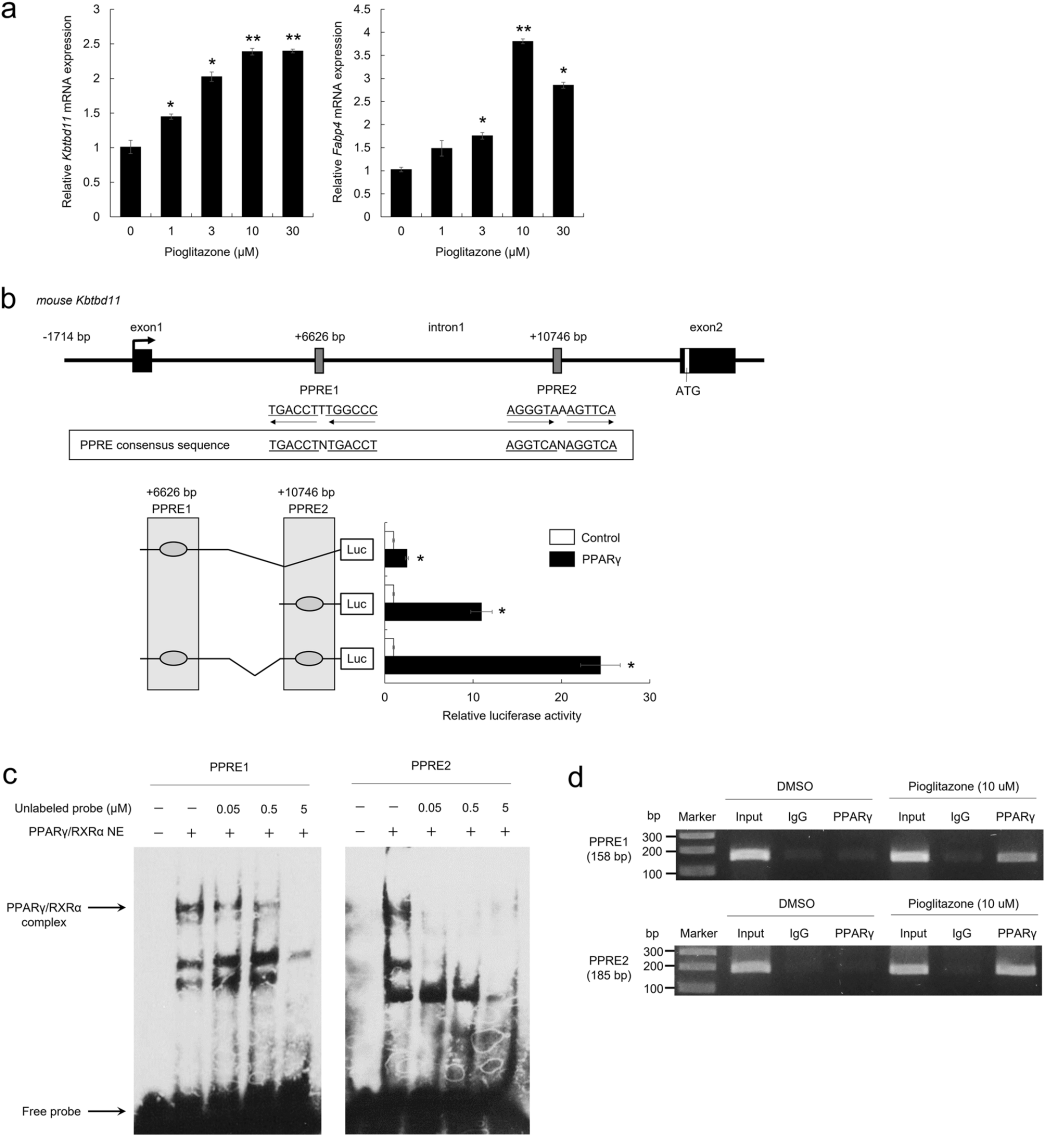
PPARγ promotes *Kbtbd11* mRNA expression. (**a**) The effect of pioglitazone on *Kbtbd11* expression in 3T3-L1 cells. *n* = 3 per group, *p < 0.05, **p < 0.01 vs. control. 3T3-L1 cells were incubated with pioglitazone (0–30 μM) for 24 h. The expression levels of *Kbtbd11* mRNA (left panel) and *Fabp4* mRNA (right panel) were measured using real-time PCR. (**b**) Schematic representation of the predicted PPARγ binding sites (PPRE1 and PPRE2). The most prominent *PPAR*γ binding sites are located in intron 1 of the *Kbtbd11* gene. Luciferase assays of the reporter gene pGL4.10 Kbtbd11-PPRE-Luc including either PPRE1 or PPRE2 and both the PPRE1 and PPRE2 constructs in the presence or absence of the PPARγ expression plasmid in 3T3-L1 cells. *n* = 3 per group, **p < 0.01 vs. control. (**c**) EMSA for PPARγ binding to PPRE1 and PPRE2 in intron 1 of the *Kbtbd11* gene. PPARγ/RXRα complex proteins were extracted from HEK293 cells co-transfected with PPARγ and RXRα expression vectors. Nuclear extracts of PPARγ/RXRα complex proteins were incubated with biotin-labeled *PPRE1* and PPRE2 probes of *Kbtbd11* intron 1. The competition was performed using unlabeled probes as competitors (0.05–5 μM). (**d**) ChIP assay for PPARγ binding to PPRE1 and PPRE2 in *Kbtbd11* intron 1 in pioglitazone-treated 3T3-L1 cells. 3T3-L1 cells were treated with 10 μM pioglitazone for 24 h. The extracted genomic DNA was subjected to immunoprecipitation using either PPARγ antibody or IgG as a negative control. The input is shown for comparison, which is an amplification derived from unprecipitated chromatin.

We previously showed that *Kbtbd11* expression is regulated by the lipid metabolism-related transcription factor USF1, suggesting that the *Kbtbd11* gene expression regulation could affect the adipose tissue^2^. Although *Kbtbd11* was suggested to be an important factor in adipogenesis, its transcriptional regulation during adipocyte differentiation remains unclear. To better understand the details of *Kbtbd11* gene expression regulation during adipocyte differentiation, we examined the *Kbtbd11* expression level in 3T3-L1 adipocytes cultured in pioglitazone, a PPARγ agonist, rather than in adipocyte differentiation cocktail (insulin, dexamethasone, and 3-isobutyl-1-methylxanthine). The results showed that pioglitazone upregulated both fatty acid binding protein 4 (*Fabp4*), the target gene of PPARγ, and *Kbtbd11* (Fig. 1a). Since pioglitazone is a PPARγ agonist, we hypothesized that it could be involved in *Kbtbd11* expression. Therefore, we used LASAGNA-Search 2.0 to analyze the potential PPARγ binding sites in the *Kbtbd11* promoter region. We identified two PPAR response element (*PPRE*) consensus sequences (PPRE1 and PPRE2) in the region containing exon 1 and intron 1 of the *Kbtbd11* gene (Fig. 1b), where PPARγ is presumed to be bound. We performed a reporter assay to examine whether PPARγ acts on this region to regulate *Kbtbd11* gene expression. When we used *Kbtbd11* promoter vectors containing either a single or both PPRE sequences in the reporter assays, both PPRE1 and PPRE2 increased *Kbtbd11* expression. In particular, PPRE2 increased *Kbtbd11* transcriptional activity, and the presence of both PPRE1 and PPRE2 synergistically increased *Kbtbd11* transcriptional activity (Fig. 1b). To examine whether PPARγ binds directly to PPREs, we performed Electrophoretic mobility shift assay (EMSA) using PPARγ/RXRα nuclear extracts and observed that PPARγ bound to both PPRE1 and PPRE2 (Fig. 1c). In addition, we examined PPARγ binding to PPREs by a chromatin immunoprecipitation (ChIP) assay in 3T3-L1 cells. Our results showed that PPARγ bound to both PPRE1 and PPRE2 in the *Kbtbd11* promoter in pioglitazone-treated 3T3-L1 cells (Fig. 1d). These results identified *Kbtbd11* as a novel PPARγ target gene.

### KBTBD11-interacting protein identification

**Figure 2:**
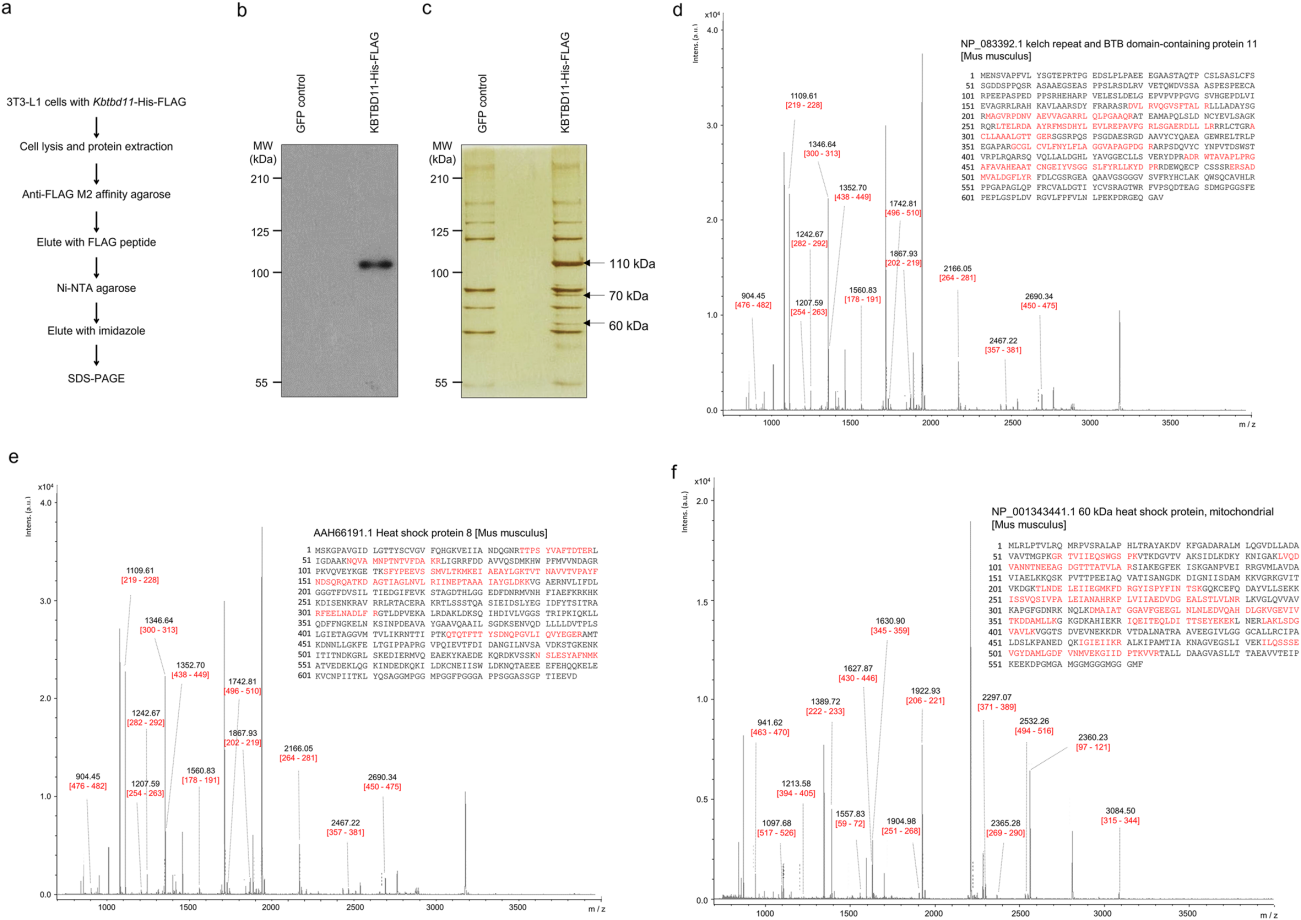
KBTBD11-interacting protein identification. (**a**) Schematic representation of the purification method of the KBTBD11-containing protein complexes using tandem affinity purification. (**b**) The KBTBD11-FLAG-His protein was immunoblotted using an anti-FLAG antibody. (**c**) The protein complexes containing KBTBD11-FLAG-His were separated using SDS-PAGE and subjected to silver staining. The samples were purified from differentiated 3T3-L1 adipocytes (day 4) infected with FLAG-His-tagged KBTBD11 adenovirus. (**d**–**f**) MALDI-TOF MS identification of the protein complexes containing KBTBD11-FLAG-His. Representative MALDI-TOF spectra of the indicated protein bands (**d**, 110 kDa; **e**, 70 kDa; **f**, 60 kDa). The identification details show the encoded proteins from Mascot searches.

We performed tandem affinity purification to identify proteins that interact with KBTBD11 in 3T3-L1 adipocytes. We infected the cells with FLAG-His-tagged KBTBD11 adenovirus, then purified and analyzed the KBTBD11-FLAG-His-containing protein complex using FLAG antibody affinity gel and nickel agarose, respectively (Fig. 2a). We immunoblotted the purified FLAG-His-tagged KBTBD11 with an anti-FLAG antibody (Fig. 2b). The ILDR2-interacting proteins were separated by SDS-PAGE and visualized by silver staining (Fig. 2c). Using matrix-assisted laser desorption/ionization time-of-flight mass spectrometry (MALDI-TOF MS), we identified the different bands of the isolated KBTBD11 complex proteins as follows: the 110, 70, and 60 kDa bands corresponded to KBTBD11 (Fig. 2d), HSC70 (Fig. 2e), and HSP60 (Fig. 2f), respectively. These results suggested that KBTBD11 interacts with HSC70 and HSP60.

### KBTBD11 interacts with HSC70 and HSP60

**Figure 3:**
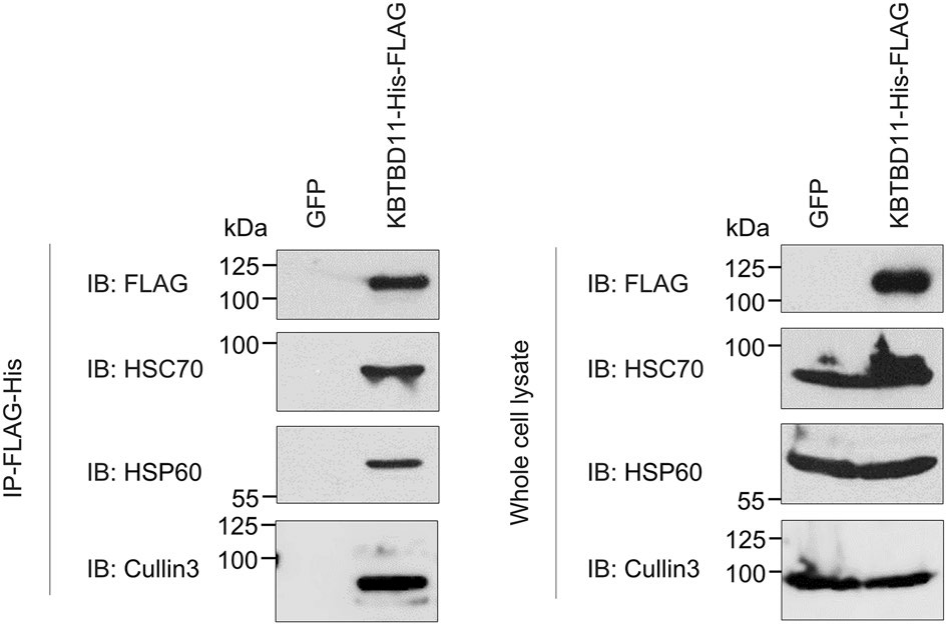
KBTBD11 interacts with HSC70 and HSP60. 3T3-L1 cells were infected either with GFP or FLAG-His-tagged *Kbtbd11* adenovirus. After the infection, whole-cell lysates were prepared and immunoprecipitated with the anti-FLAG antibody, followed by immunoblotting with anti-FLAG, anti-HSC70, anti-HSP60, and anti-Cullin3 antibodies.

To determine whether KBTBD11 physically binds to HSC70 and HSP60 in the cells, we performed co-immunoprecipitation. We infected either GFP or FLAG-His-tagged KBTBD11 adenovirus into 3T3-L1 cells and examined the binding of KBTBD11-FLAG-His to endogenous HSC70 and HSP60. After the KBTBD11 transduction, we prepared whole-cell lysates and immunoprecipitated them with appropriate anti-FLAG antibodies. The results confirmed that KBTBD11 binds directly to endogenous HSC70 and HSP60 (Fig. 3). KBTBD11 reportedly interacted with Cullin3^5^; thus, we examined whether KBTBD11 interacts with Cullin3 as well as HSC70 and HSP60. Our results supported the prior study and showed that KBTBD11 interacts with Cullin3 (Fig. 3). To determine whether Cullin3 binds to HSC70 and HSP60 in the cells, we performed co-immunoprecipitation. We then infected either GFP or FLAG-His-tagged KBTBD11 adenovirus into 3T3-L1 cells and examined the binding of Cullin3 to HSC70 and HSP60. After the KBTBD11 transduction, we prepared whole-cell lysates and immunoprecipitated them with appropriate anti-Cullin3 antibodies. The results confirmed that Cullin3 binds to KBTBD11. In addition, Cullin3 bound to HSC70 but not to HSP60 (Supplementary Fig. S1). Our results suggested that direct Cullin3 binding to HSC70 was present.

### HSC70 and HSP60 affect KBTBD11 protein levels

**Figure 4:**
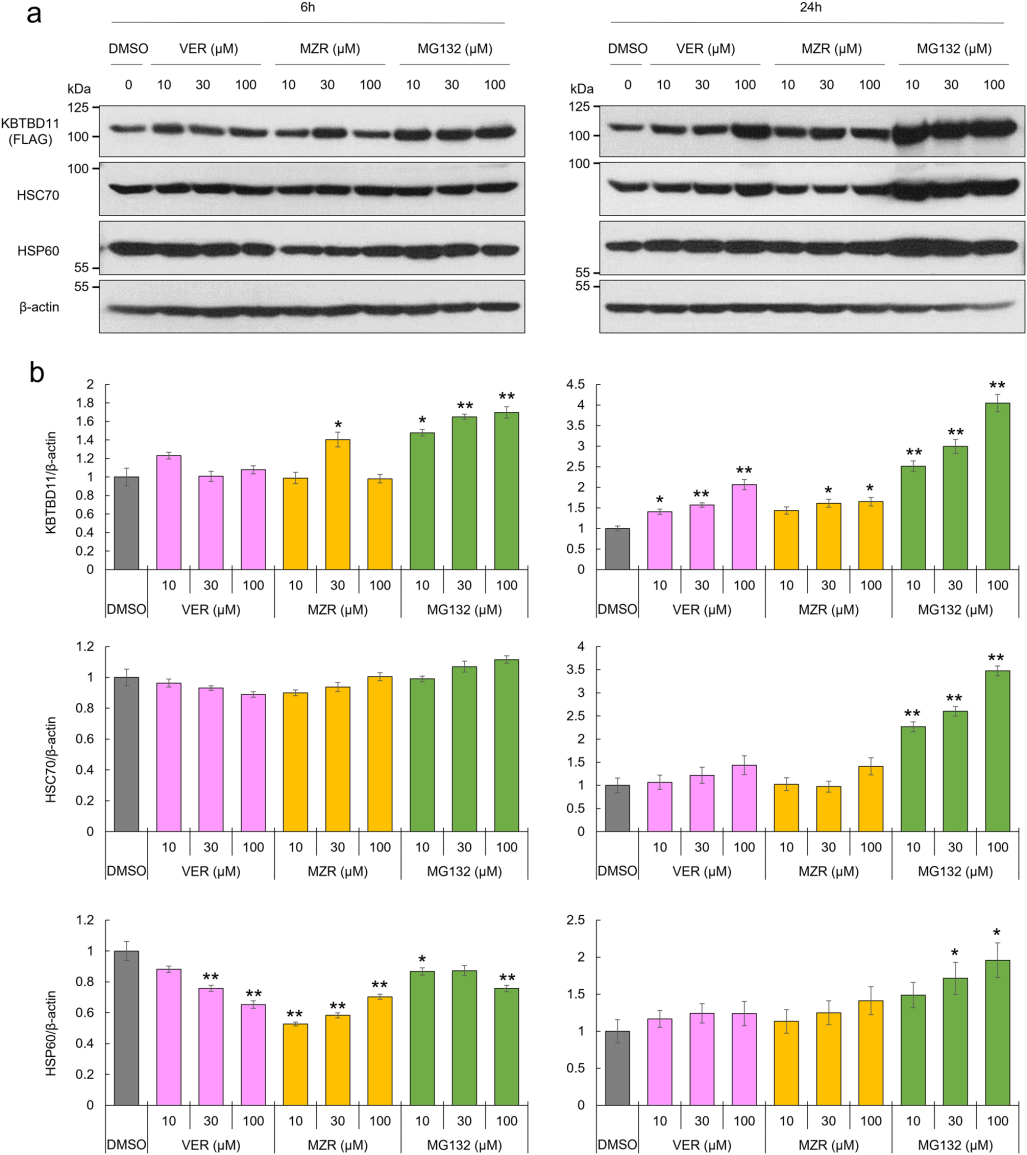
HSC70 and HSP60 affect KBTBD11 protein levels. (**a**) 3T3-L1 cells were infected with FLAG-His-tagged *Kbtbd11* adenovirus. After 48 h of infection, incubated for 6 and 24 h with or without various VER, MZR, and MG132 concentrations, whole-cell lysates were prepared and subjected to western blot analysis using anti-FLAG, anti-HSC70, anti-HSP60, and β-actin antibodies. (**b**) Quantification of the relative KBTBD11, HSC70, and HSP60 protein levels detected by western blot. The data were normalized to β-actin. *n* = 3 per group, *p < 0.05, **p < 0.01 vs. DMSO control.

Both HSC70 and HSP60, heat shock proteins, are involved in protein folding and stability in cells^6,7^. Since we hypothesized that KBTBD11 binding to HSC70 and HSP60 could affect KBTBD11 protein folding or stability, we investigated how HSC70 and HSP60 inhibitors affect KBTBD11 protein levels. To examine how HSC70 and HSP60 inhibitors could affect KBTBD11 protein levels, we supplemented KBTBD11-expressing 3T3-L1 cells with the HSC70 inhibitor VER-155008 (VER), the HSP60 inhibitor mizoribine (MZR), and the proteasome inhibitor MG132, then confirmed the KBTBD11 protein expression by western blotting. VER-treated KBTBD11-expressing 3T3-L1 cells exhibited a concentration-dependent increase in the KBTBD11 protein level at 24 h compared to the DMSO-treated control (Fig. 4a,b). The MZR-treated KBTBD11-expressing 3T3-L1 cells showed increased KBTBD11 protein levels at both 6 h and 24 h compared to the control (Fig. 4a,b). To examine whether different inhibitors of HSC70 and HSP60 alter KBTBD11 protein levels, we examined KBTBD11 protein levels using the HSC70 inhibitor Apoptozole and the HSP60 inhibitor Gossypol. Both Apoptozole and Gossypol-treated KBTBD11-expressing 3T3-L1 cells also increased KBTBD11 protein levels at 24 h compared to the DMSO-treated control, similar to VER and MZR (Supplementary Fig. S2). Finally, the MG132-treated KBTBD11-expressing 3T3-L1 cells displayed a concentration-dependent increase in the KBTBD11 protein level at both 6 h and 24 h compared to the control (Fig. 4a,b). The HSC70 protein level remained unchanged under all inhibitor treatment conditions at 6 h, although it significantly increased upon the MG132 treatment at 24 h. HSP60 levels significantly decreased under all three inhibitor conditions at 6 h compared to the control (Fig. 4a,b). Interestingly, MG132 significantly increased the HSP60 level at 24 h. Therefore, the HSC70 and HSP60 inhibition-related KBTBD11 increase suggested that HSC70 and HSP60 could be involved in the KBTBD11 protein stability or degradation.

### The involvement of HSC70 and HSP60 in KBTBD11 stability and NFATc1 ubiquitination

**Figure 5:**
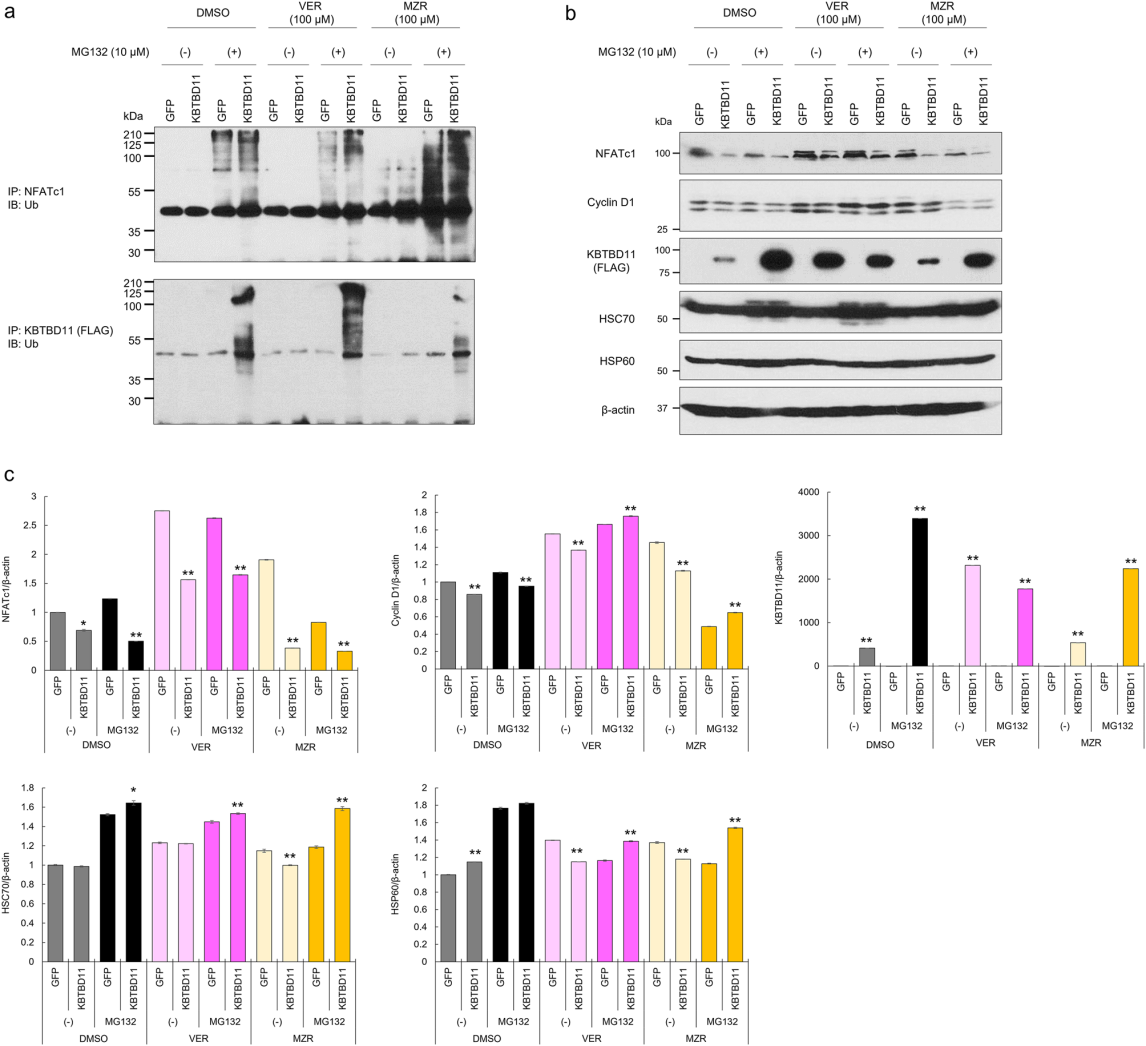
HSC70 and HSP60 involvement in KBTBD11 stability and NFATc1 ubiquitination. 3T3-L1 cells were infected either with GFP or FLAG-His-tagged *Kbtbd11* adenovirus. After 48 h of infection, incubated for 24 h with or without 100 μM VER, 100 μM MZR, and 10 μM MG132. (**a**) Whole-cell lysates were prepared and immunoprecipitated using an anti-FLAG antibody, followed by immunoblotting with anti-ubiquitin and anti-FLAG antibodies. (**b**) Whole-cell lysates were subjected to western blot analysis using anti-NFATc1, anti-Cyclin D1, anti-FLAG, anti-HSC70, anti-HSP60, and β-actin antibodies. (**c**) Quantification of the relative NFATc1, Cyclin D1, KBTBD11, HSC70, and HSP60 protein levels detected by western blot. The data were normalized to β-actin. n = 3 per group, *p < 0.05, **p < 0.01 vs. GFP control.

We demonstrated that the *Kbtbd11* gene is involved in adipocyte differentiation^1^, although the role of the KBTBD11 protein in adipocytes remains elusive. In osteoclasts, KBTBD11 reportedly interacts with Cullin3 to regulate NFATc1 ubiquitination^5^. Therefore, we examined how the HSC70- and HSP60-mediated KBTBD11 protein level regulation affects NFATc1 ubiquitination. We supplemented KBTBD11-expressing 3T3-L1 cells either with HSC70 or HSP60 inhibitors with or without MG132, then examined the KBTBD11 and NFATc1 protein levels. We observed that NFATc1 ubiquitination was promoted by the KBTBD11 increase upon both HSC70 and HSP60 inhibition compared to the GFP controls (Fig. 5a). Consistent with this result, NFATc1 protein levels were reduced in KBTBD11-expressing cells compared to the GFP controls (Fig. 5b). We also examined KBTBD11 ubiquitination and observed that HSC70 inhibition caused an increase in KBTBD11 ubiquitination compared to the GFP control, while HSP60 inhibition decreased ubiquitination of KBTBD11 (Fig. 5a). To examine the NFATc1 target factor, we examined the protein levels of Cyclin D1, as its encoding gene is an NFATc1 target. We detected reduced Cyclin D1 protein levels with decreasing NFATc1 protein levels upon KBTBD11 increase. Consistent with Fig. 4, KBTBD11 protein levels increased upon both HSC70 and HSP60 inhibition without MG132 compared to the GFP control (Fig. 5b). The MG132 treatment increased the HSC70 protein levels in both GFP- and KBTBD11-expressing cells (Fig. 5b,c). These results suggested that KBTBD11 protein level changes by both HSC70 and HSP60 inhibitors regulate NFATc1 ubiquitination and affect NFATc1 protein levels in the cells and these series of events might affect NFATc1-mediated Cyclin D1 levels hence cell proliferation.

## Discussion

In this study, we demonstrated that the *Kbtbd11* is a novel target gene of PPARγ (Fig. 1), which is an important master regulator of adipocyte differentiation and regulates the transcription of genes involved in adipogenesis, such as *Fabp4* and *CD36*. Therefore, the PPARγ target gene, *Kbtbd11*, may regulate adipocyte differentiation and adipogenesis. We have previously reported that *Kbdbd11* is upregulated during 3T3-L1 adipocyte differentiation, its knockdown inhibits adipocyte differentiation, whereas *Kbtbd11* overexpression promotes it^1^. These results strongly suggest that *Kbtbd11* is a target gene of PPARγ.

*KBTBD11* is involved in both adipocyte and osteoclast differentiation^1,5^, and it is associated with colorectal cancer cell susceptibility^8^, although the functions of KBTBD11 are not fully understood. Therefore, we aimed to investigate the role of KBTBD11 proteins in adipocytes, we thus looked for KBTBD11 interacting proteins and identified HSC70 and HSP60 (Fig. 2).

Heat shock proteins (HSPs) play an important role in preventing stress-cell damage caused by various stresses through the proper protein folding and degradation of intracellular proteins^9–11^. HSC70, a member of the HSP family, is a cytoplasmic protein abundantly and ubiquitously expressed in most cells^12^. HSC70 is involved in various important functions as a molecular chaperone that promotes the correct folding of nascent polypeptides and proteins for degradation^6^, as well as for protein homeostasis or synthesis, and other essential intracellular activities^13^. In addition, HSC70 also binds to the carboxyl terminus of the Hsp70-interacting protein (CHIP), an E3 ligase^10^. The complex properly ubiquitinates client proteins bound to HSC70 and facilitates their proteasome-mediated degradation^14^. Therefore, the molecular chaperone HSC70 promotes newly synthesized protein folding and it is also involved in protein degradation. The extent to which the opposing effects of HSC70, protein folding and proteolysis, are regulated is unknown. In our results, HSC70 inhibition increased intracellular KBTBD11 protein levels (Figs. 4, 5), suggesting that KBTBD11-HSC70 binding normally promotes KBTBD11 degradation. In the HSC70 inhibitor VER group with MG132 (Fig. 5a), KBTBD11 ubiquitination was promoted. However, this increased KBTBD11 ubiquitination might be due to the control of excess KBTBD11 in the cells upon HSC70 inhibition or increased KBTBD11 misfolding.

Another heat shock protein, HSP60, which we identified in this study to bind KBTBD11, plays an important role in protein homeostasis by mediating protein folding and assembly as a molecular chaperone^7^. HSP60 classically maintains protein homeostasis in the organelles as a mitochondrial resident^15^. HSP60 is also present not only inside mitochondria, but also in other cellular locations, including the cytoplasm and extracellular space, and its function varies depending on the interacting factors that surround it at each location^7^. For example, in the mitochondria, HSP60 contributes to the folding and transport of other proteins, while in the cytoplasm, it promotes apoptosis in certain cancer cells or, conversely, an anticancer agent^16,17^. In addition, HSFP60 interacts with cytoplasmic proteins and regulates the stability and degradation of those proteins. For example, the p53-mediated blocking of the HSP60 complex increases p53 stability, while blocking HSP60 in complex with Survivin results in Survivin protein degradation^18^. Therefore, HSP60 reportedly regulates apoptosis through two mechanisms: Survivin stability and p53 protein regulation^17^. The results of this study suggested that KBTBD11 stabilization by the HSP60 inhibitor-mediated disruption of the KBTBD11-HSP60 complex might have reduced KBTBD11 ubiquitination and increased intracellular KBTBD11 levels (Fig. 5a). Therefore, the interaction between the HSP60 protein and KBTBD11 might be involved in the degradation of the KBTBD11 protein.

The results of this study showed that both HSC70 and HSP60 inhibitors increased KBTBD11 protein levels; this consequently reduced NFATc1 (Fig. 5b). However, expression level of NFATc1 was not reduced by treatment with VER or MZR compared with DMSO in cells untreated with MG132. In contrast, KBTBD11 protein level was not increased by treatment with VER or MZR in cell treated with MG132 (Fig. 5b). Although the cause is not clear, it is possible that the effect of the simultaneous treatment of MG132 with VER or MZR affected KBTBD11 protein levels. Since MG132, a proteasome inhibitor, inhibits NFATc1 degradation, we speculated that the expression level of NFATc1 might not be altered in MG132-treated KBTBD11-overexpressing cells compared with MG132-treated GFP-expressing cells. However, the expression level of NFATc1 was decreased in MG132-treated KBTBD11-overexpressing cells compared with MG132-treated GFP-expressing cells (Fig. 5b). This result may be attributable to the fact that the amount of NFATc1 protein was already decreased by KBTBD11 overexpression before administration of reagents (HSC70, HSP60, and MG132). Since KBTBD11 overexpression has been reported to promote NFATc1 ubiquitination in osteoclasts^5^, it is possible that KBTBD11 overexpression also promoted NFATc1 ubiquitination in 3T3-L1 cells, leading to a decrease in NFATc1 protein levels.

NFATc1 is required for adequate osteoclast differentiation, in particular, acting there as a master transcriptional regulator of osteoclast differentiation^19^. In addition, constitutively active NFATc1 disrupts the mechanisms that regulate physiological cell proliferation and differentiation in 3T3-L1 cells, transforming these immortalized cells and turning them into cancer cells^20^. Therefore, NFATc1 is involved in cell differentiation and might be involved in adipocyte differentiation by affecting cell proliferation, especially in 3T3-L1 cells during the early differentiation stages from preadipocytes to mature adipocytes.

Upon supplementation with an adipocyte differentiation inducer, the growth-arrested 3T3-L1 preadipocytes synchronously re-enter the cell cycle and undergo one to two cell divisions. This phenomenon, called MCE, is an important early event in 3T3-L1 adipocyte differentiation. Cyclin D1 is an important sensor for this division, promoting the G1-to-S phase transition in the cell cycle progression^2^. In addition, since *cyclin D1* is a target gene of NFATc1^21^, the KBTBD11-NFATc1-CyclinD1 signaling could be potentially important in understanding MCE during 3T3-L1 adipocyte differentiation. In our previous study^1^, *Kbtbd11* knockdown reduced *cyclin D1* gene expression and suppressed MCE along with 3T3-L1 adipocyte differentiation, while *Kbtb11* overexpression increased *cyclin D1* gene expression, promoted MCE, and enhanced 3T3-L1 adipocyte differentiation. These results suggested that *Kbtbd11* plays an important role in MCE during 3T3-L1 adipocyte differentiation.

Increased KBTBD11 protein levels decreased NFATc1 as well as Cyclin D1 (Fig. 5b). As *Kbtbd11* overexpression induces MCE along with an increase in *cyclin D1* expression^1^, Cyclin D1 reduction coupled with an increased KBTBD11 protein levels upon using both HSC70 and HSP60 inhibitors might seemingly not promote MCE. NFATc1 is a *cyclin D1-*regulating transcription factor^21^. However, the Cyclin D1 reduction is not only due to the HSC70 and HSP60 inhibitor-related KBTBD11 expression increase and the associated NFATc1 reduction. HSC70 promotes Cyclin D1 protein synthesis^22^ and HSP60 increases *cyclin D1* transcription via β-catenin^23^. Therefore, the KBTBD11-NFATc1-Cyclin D1 effector mechanism, as well as these HSC70 and HSP60 inhibitors, could be directly involved in Cyclin D1 protein synthesis. Therefore, the aforementioned HSC70/HSP60-KBTBD11-NFATc1-Cyclin D1 effector mechanism would require a more detailed future investigation.

**Figure 6:**
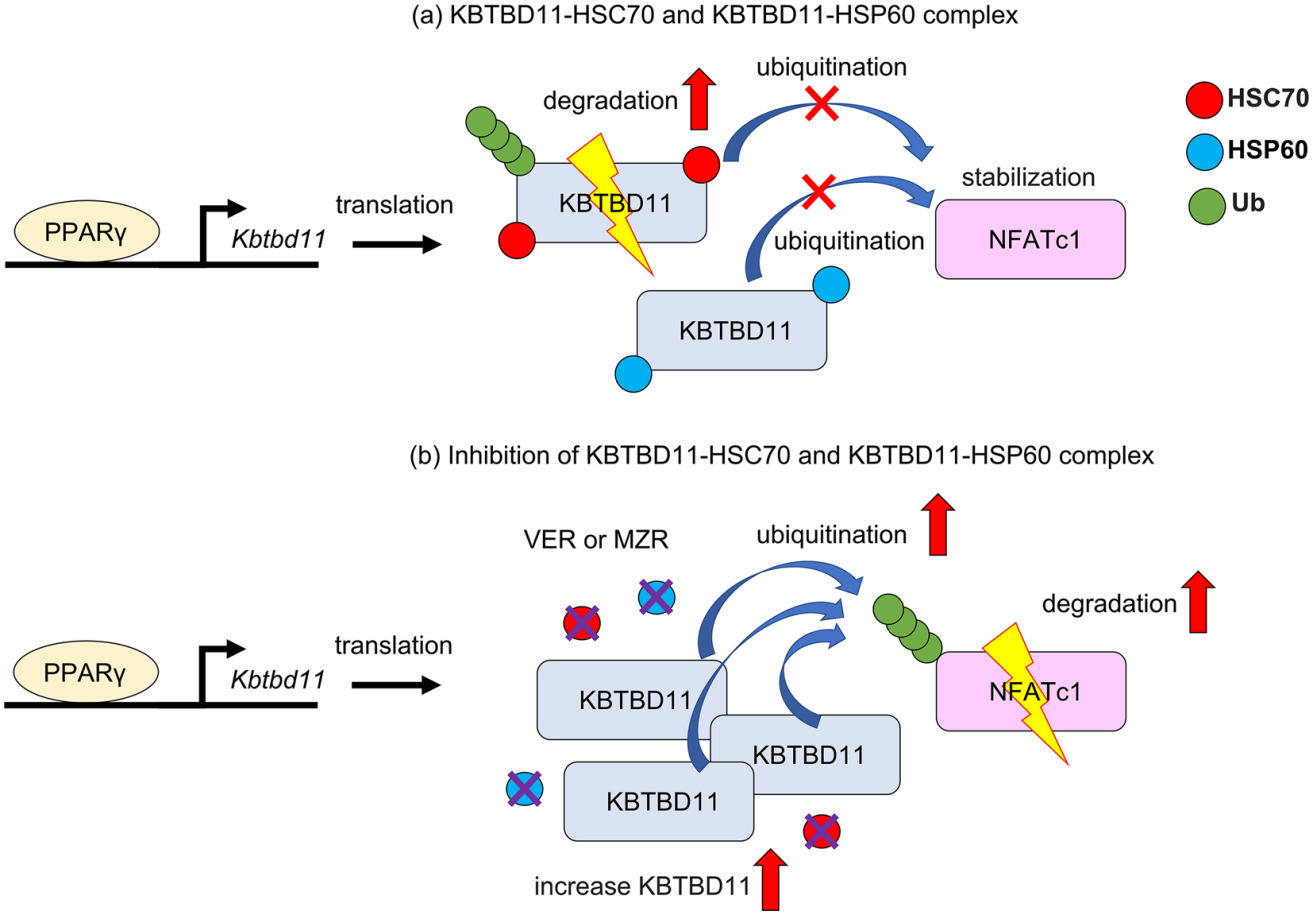
Schematic representation of the relationship between KBTBD11 and NFATc1 degradation in 3T3-L1 cells. During adipocyte differentiation, the increase in PPARγ is accompanied by an increased expression of the target gene, *Kbtbd11*. Next, *Kbtbd11* is translated into KBTBD11, which binds to HSC70 and HSP60. The HSC70 and HSP60 binding to KBTBD11 is involved in the maintenance of the intracellular KBTBD11 protein levels and that of its target NFATc1 for proteolysis. (**a**) Under normal conditions, HSC70 and HSP6 maintain intracellular KBTBD11 protein homeostasis, which allows stable NFATc1 function. (**b**) The dissociation of the KBTBD11-HSC70 or KBTBD11-HSP60 complexes upon HSC70 and HSP6 inhibition might be responsible for the stability of KBTBD11 and increase the amount of KBTBD11 in the cell. As a result, the increased KBTBD11 ubiquitinates NFATc1 and promotes a decrease in intracellular NFATc1 levels.

In summary, this study identified the KBTBD11-encoding gene as a novel PPARγ target. Moreover, we described that the KBTBD11 protein interacts with HSC70 and HSP60, which are involved in the degradation and stabilization of the KBTBD11 protein. KBTBD11 protein levels affect NFATc1 degradation by NFATc1 ubiquitination and stabilization, which might be involved in early adipocyte proliferation and differentiation (Fig. 6). The future elucidation of the HSC70/HSP60-KBTBD11 interaction-related NFATc1 protein regulation could pave the way to potential proliferation- and differentiation-related applications not only in adipocytes but also in various other cell types, including osteoclasts and cancer cells.

## Materials and methods

### Animals

No animals were used in this study.

### Chemicals

VER-155008, Mizoribine, and MG132 were purchased from Cayman Chemicals, Tokyo Chemical Industry, and Chemscene, respectively. Apoptozole and Gossypol were purchased from MedChemExpress LLC.

### Cell culture

293A cells (Thermo Fisher Scientific, R70507) were maintained in high-glucose Dulbecco’s modified Eagle medium (DMEM) supplemented with 10% fetal bovine serum (FBS) and 100 units of both penicillin and streptomycin at 37 °C in 5% CO_2_. 3T3-L1 cells (National Institutes of Biomedical Innovation, Health and Nutrition, JCRB9014) were maintained in low-glucose DMEM supplemented with 10% FBS, and 100 units of both penicillin and streptomycin at 37 °C in 5% CO_2_. 3T3-L1 adipocyte differentiation was performed as described previously^24,25^. Briefly, 3T3-L1 cells were cultured in high-glucose DMEM until confluence. The medium was then replaced with high-glucose DMEM containing 5 μg/mL insulin, 1 μM dexamethasone, and 0.5 mM 3-isobutyl-1-methylxanthine. The medium was replaced 48 h later with high-glucose DMEM containing 5 μg/mL insulin. The medium was then renewed every 2 days.

### Real-time PCR (qPCR)

Total RNA and cDNA were prepared as described previously^2^. The qPCR assays were performed using the Kapa SYBR fast universal qPCR kit (Kapa Biosystems) and the ViiA7 Real-Time PCR System. Supplementary Table 1 lists the primers used in this study.

### Transfection and luciferase assays

Luciferase assays were performed using the Dual-Luciferase Reporter Assay System (Promega). The region containing exon 1 and intron 1 of the *Kbtbd11* gene was PCR-amplified using mouse genomic DNA as a template. 3T3-L1 cells were co-transfected with each expression vector, mouse *Kbtbd11* promoters that drive firefly luciferase expression (pGL4.10 mouse *Kbtbd11*-1714) and *Renilla reniformis* luciferase vector (pGL4.74) as an internal control reporter. The cells were incubated for 24 h post-transfection and lysed in a lysis buffer (Promega). The lysate was used for the luciferase assay and luminescence was detected using a Luminometer (Thermo Fisher Scientific).

### EMSA

The probes were synthesized using the biotin 3’ End DNA Labeling Kit (Thermo Fisher Scientific). HEK293T cells (ATCC, CRL-3216) were co-transfected with PPARγ and RXRα expression vectors. Nuclear protein extracts were prepared 48 h post-transfection. The biotin-labeled probe was mixed with the PPARγ/RXRα-overexpressing nuclear protein extract and incubated for 20 min. For competition, unlabeled excess probes (0.05–5 μM) were mixed with biotin-labeled probes and PPARγ/RXRα overexpressed nuclear protein extracts and incubated for 20 min. We used the LightShift Chemiluminescent EMSA kit (Thermo Fisher Scientific) for detection. The EMSA probes are listed in Supplementary Table 1.

### ChIP assay

For the ChIP assay, we used the SimpleChIP Enzymatic chromatin IP Kit (Cell Signaling Technology). We performed the immunoprecipitation using PPARγ antibody and mouse IgG as a negative control. After the immunoprecipitation, the associated DNA was amplified using the primers listed in Supplementary Table 1.

### Adenovirus preparation

We subcloned the C-terminal 3xFLAG- and 6xHis-tagged full-length *Kbtbd11* into the pENTR/D-TOPO vector (Thermo Fisher Scientific). The inserts of the pENTR-*Kbtbd11*-3xFLAG-6xHis vector were transferred into the pAd/CMV/V5-DEST vector using the Gateway system (Thermo Fisher Scientific). Adenoviruses were prepared and purified using the ViraPower Adenoviral Expression System (Thermo Fisher Scientific) and the Adenovirus Purification Miniprep Kit (Cell Biolabs), respectively, as described previously^26^.

### Tandem affinity purification and identification of KBTBD11-interacting proteins

The method of tandem affinity purification was described previously^26,27^. Briefly, 3T3-L1 cells infected with the adenovirus vector encoding 3xFLAG-6xHis-tagged KBTBD11 proteins were collected and suspended in the lysis buffer*.* The lysates were centrifuged, the supernatants were loaded into the anti*-*FLAG M2 agarose gel (Merck), and rotated at 5 rpm for 1 h to bind the proteins. The resin was washed with wash buffer and the bound proteins were eluted with an elution buffer containing 150 μg/mL 3xFLAG peptide. The eluted proteins were then loaded into an Ni–NTA agarose gel (Qiagen) and rotated. The resin was washed with a wash buffer, then the KBTBD11 protein complexes were eluted with an elution buffer. We performed the KBTBD11-interacting protein identification using MALDI-TOF MS, Autoflex (Bruker Daltonics), and the detected masses and peptide sequences were analyzed using the Mascot software (Matrix Science).

### Western blot

Whole-cell lysates were separated using SDS-PAGE and transferred onto PVDF membranes. The proteins were detected with HSC70 (sc7298), HSP60 (sc-13115), CUL-3 (sc-166110), NFATc1 (sc-7294), Cyclin D1 (sc-8396), and Ub (sc-8017) (Santa Cruz Biotechnology), FLAG (F1804, Merck), and β-actin antibody (G043, ABM). The blots were cropped and the full image of the non-crop blots were presented in Supplementary Figures.

### Statistical analysis

The statistical significance was tested using one-way ANOVA with the Bonferroni test for multiple comparisons. The data were expressed as the mean ± SEM. The statistical significance was set at *p* < 0.05.

## Supplementary Information

Supplementary Figures.

Supplementary Legends.

Supplementary Table 1.
