# Supplementary figures and images for: KBTBD11, encoding a novel PPARγ target gene, is involved in NFATc1 proteolysis by interacting with HSC70 and HSP60
Source: Sci Rep. 2022 Nov 24;12:20273. doi: 10.1038/s41598-022-24929-5 (PMC9700792; doi:10.1038/s41598-022-24929-5)

Figure S1

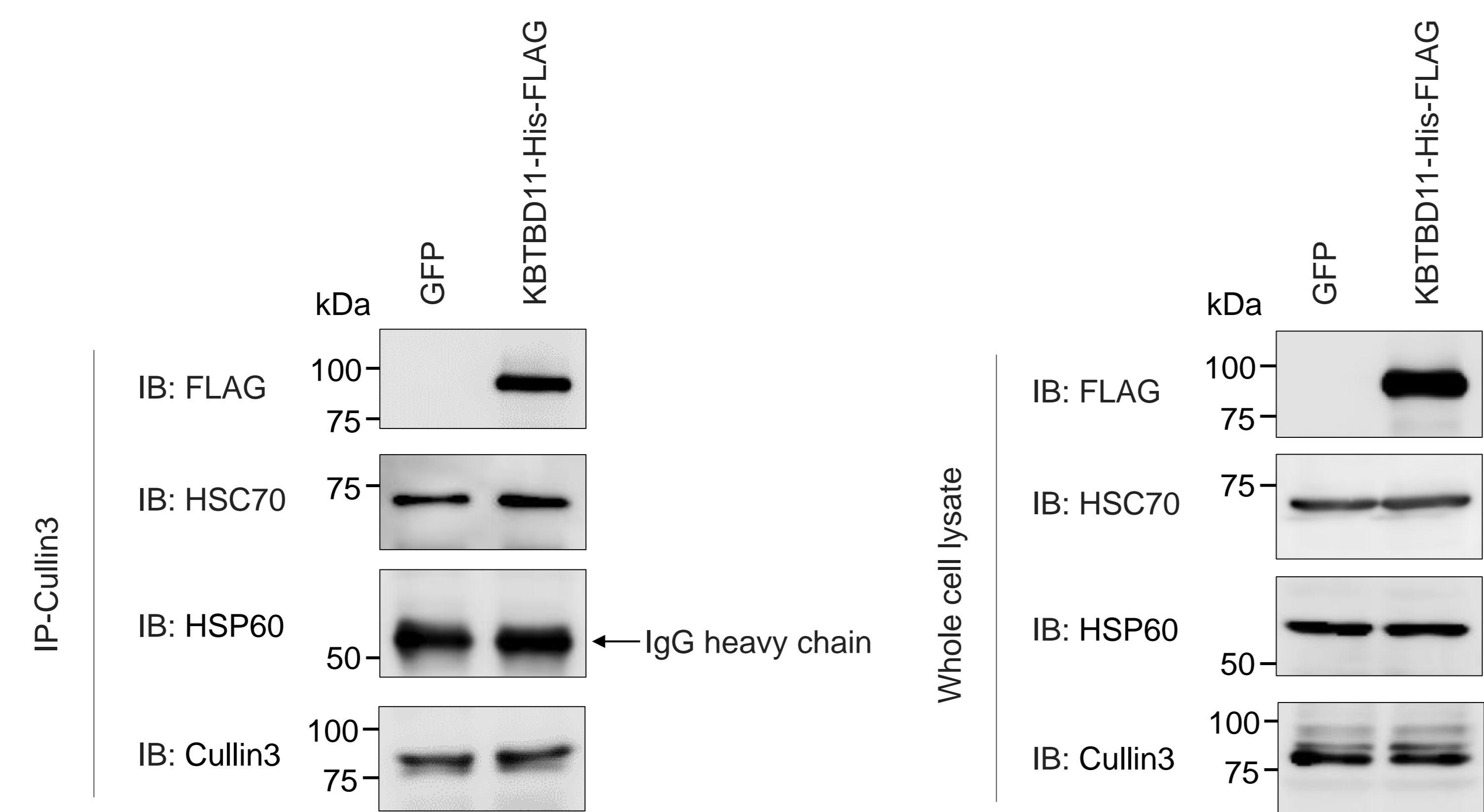

Figure S2

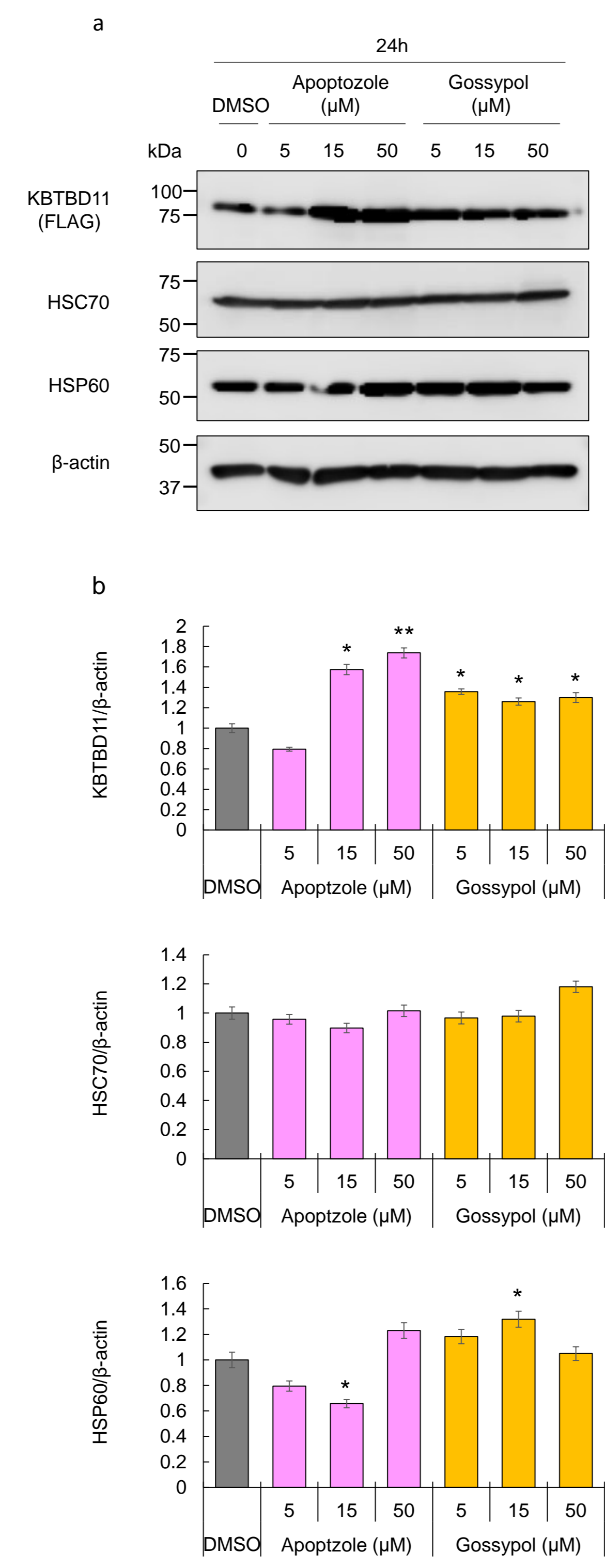

Figure S3

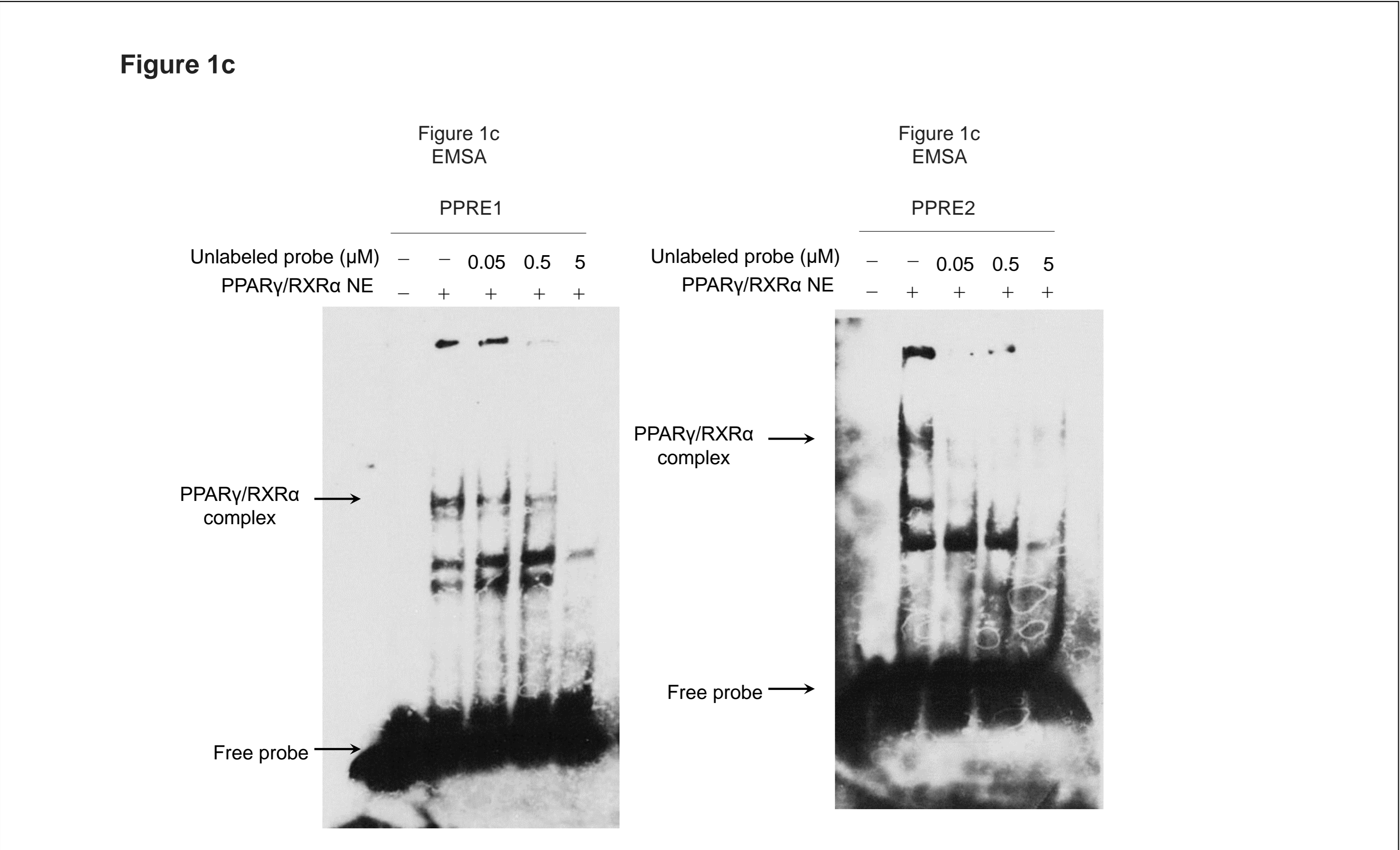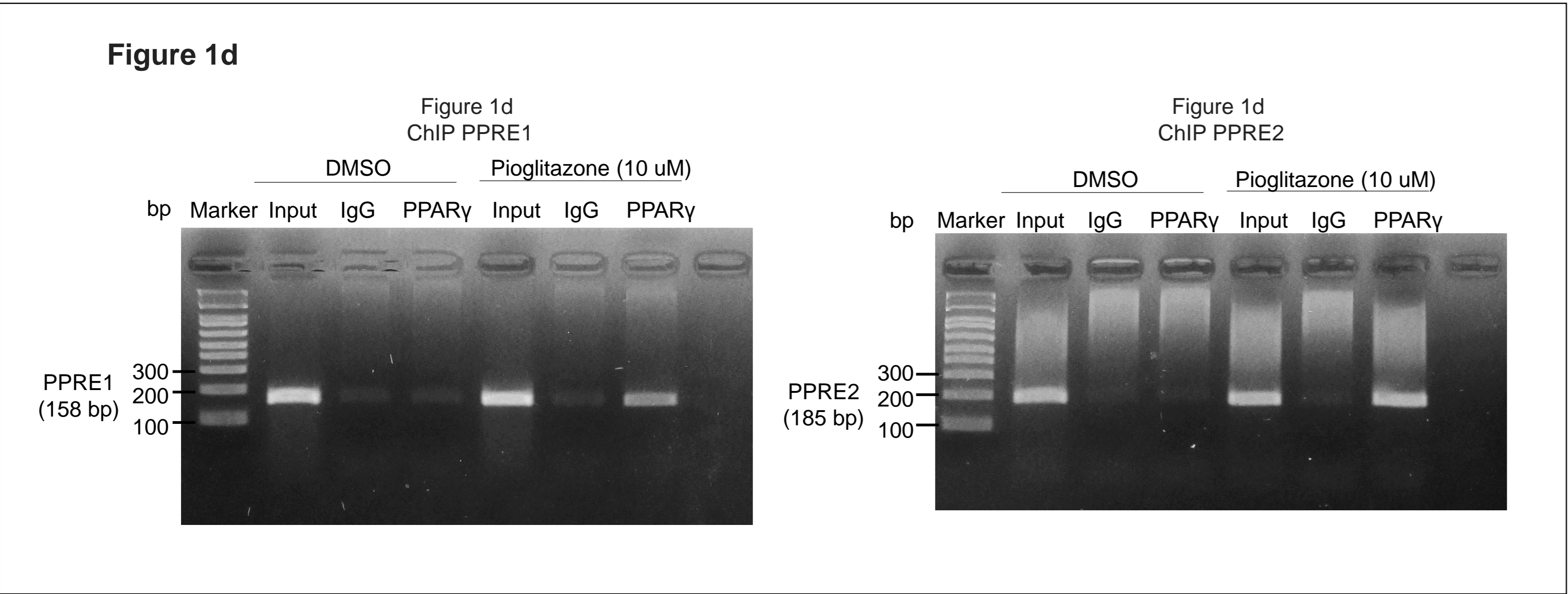

Figure S4

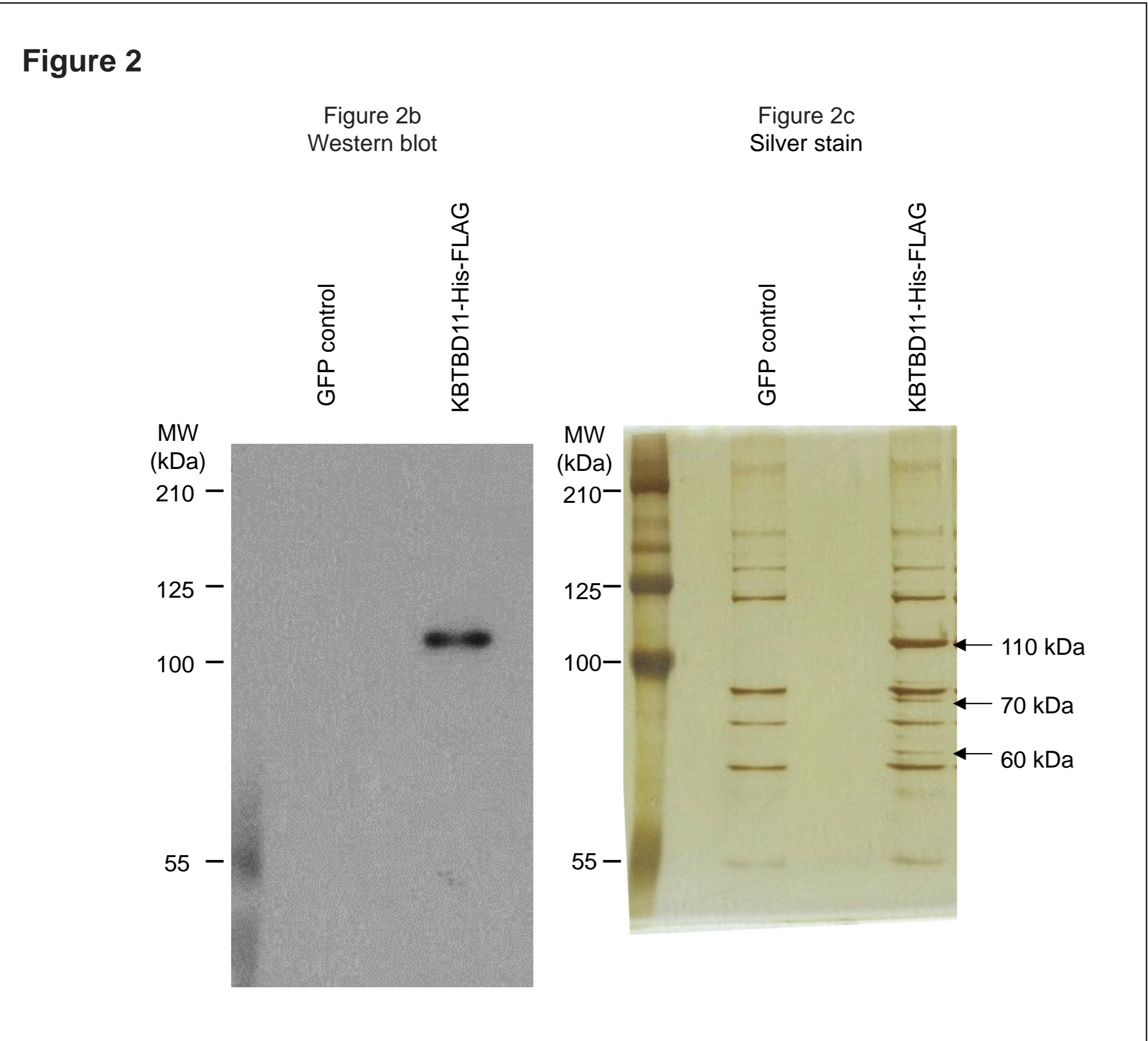

Figure S5

Figure 3

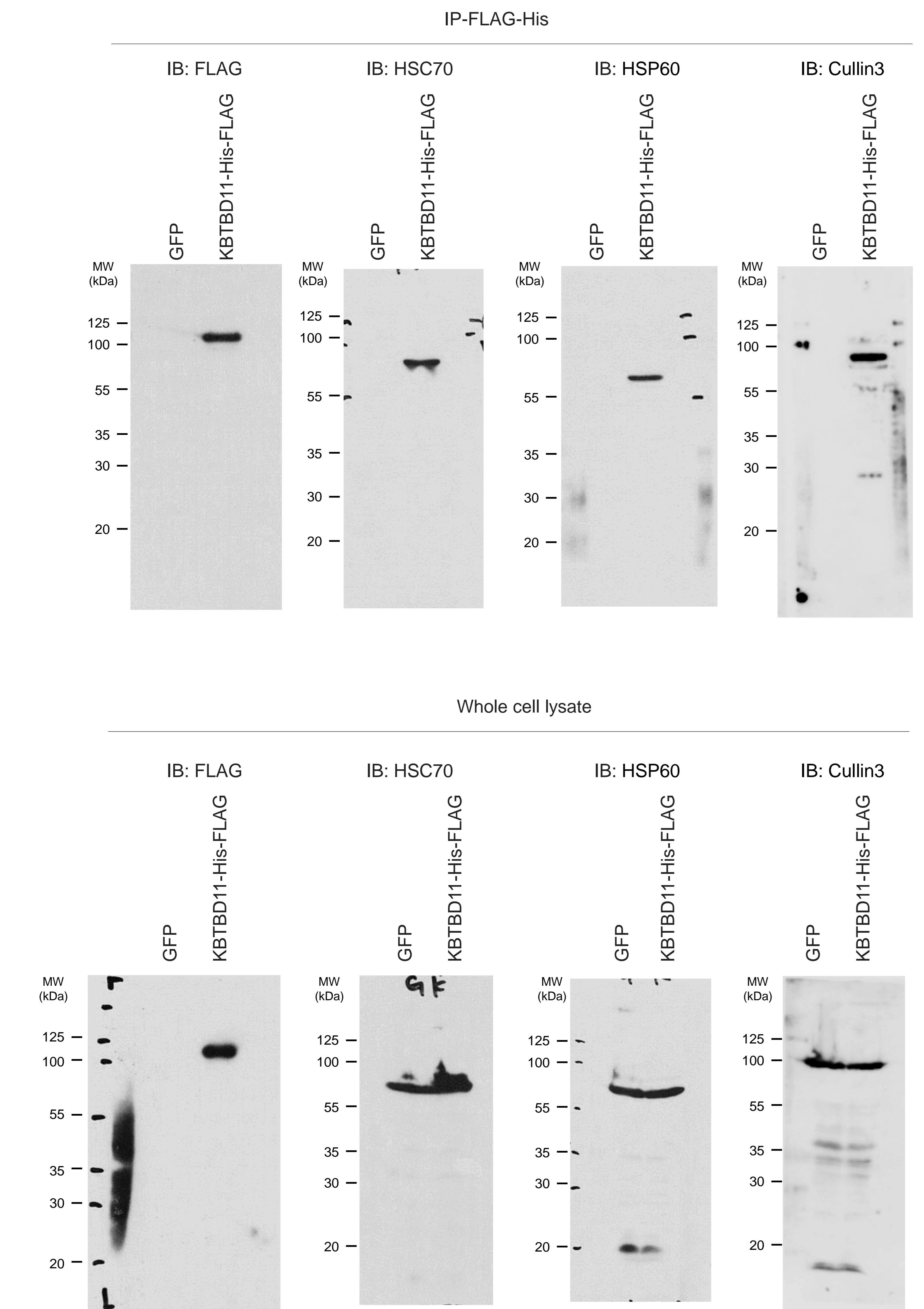

Figure S6

Figure S1

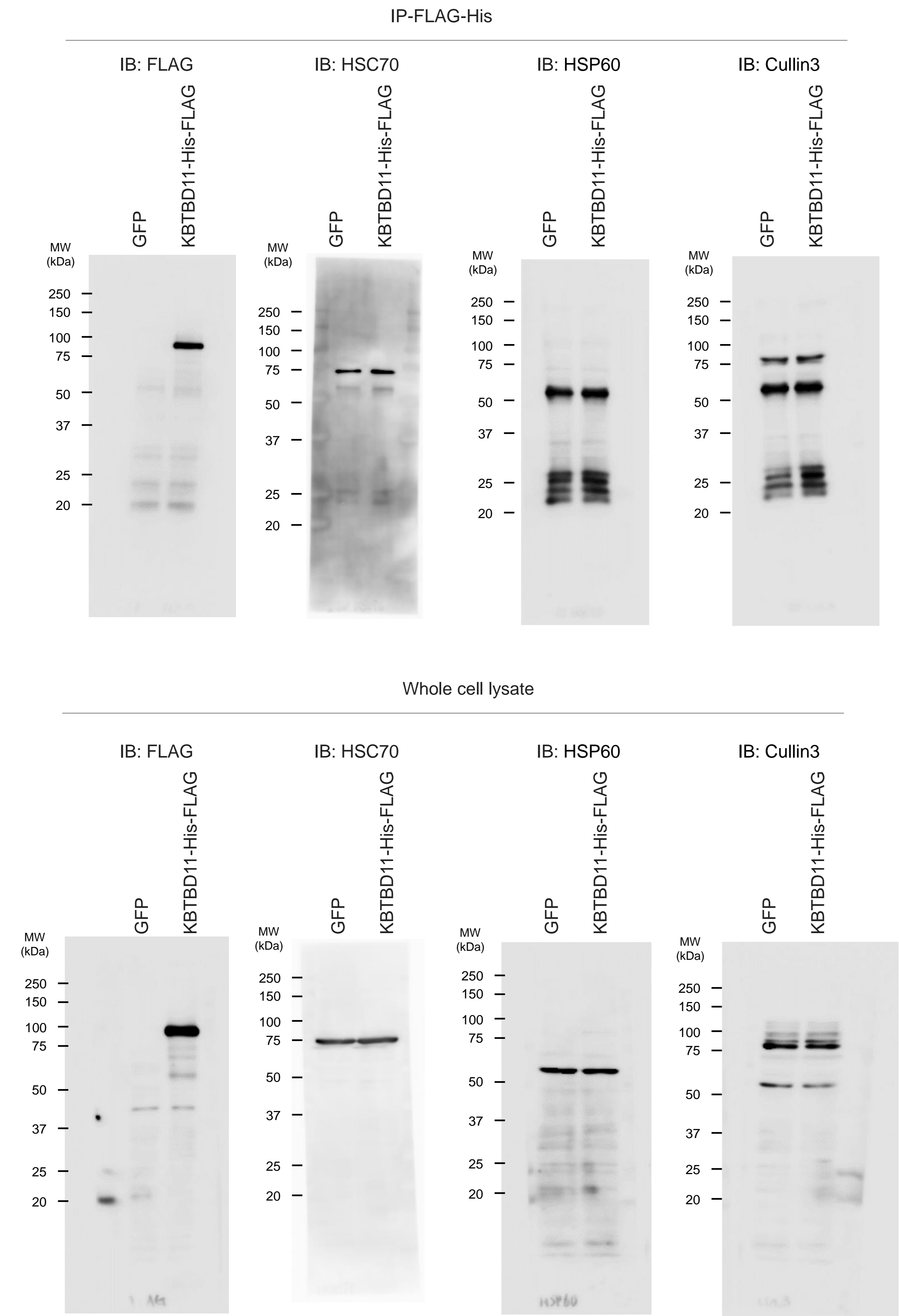

Figure S7

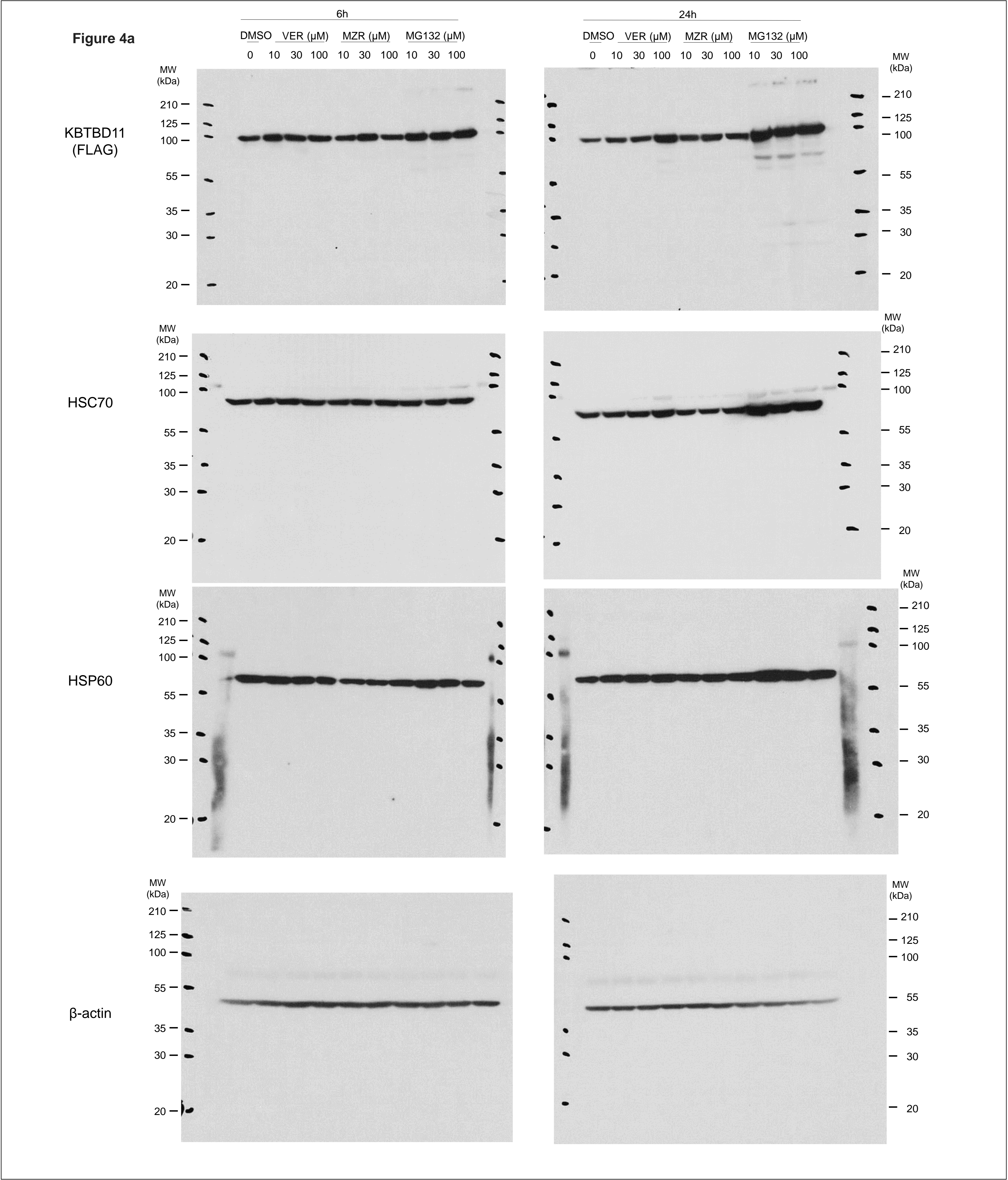

Figure S8

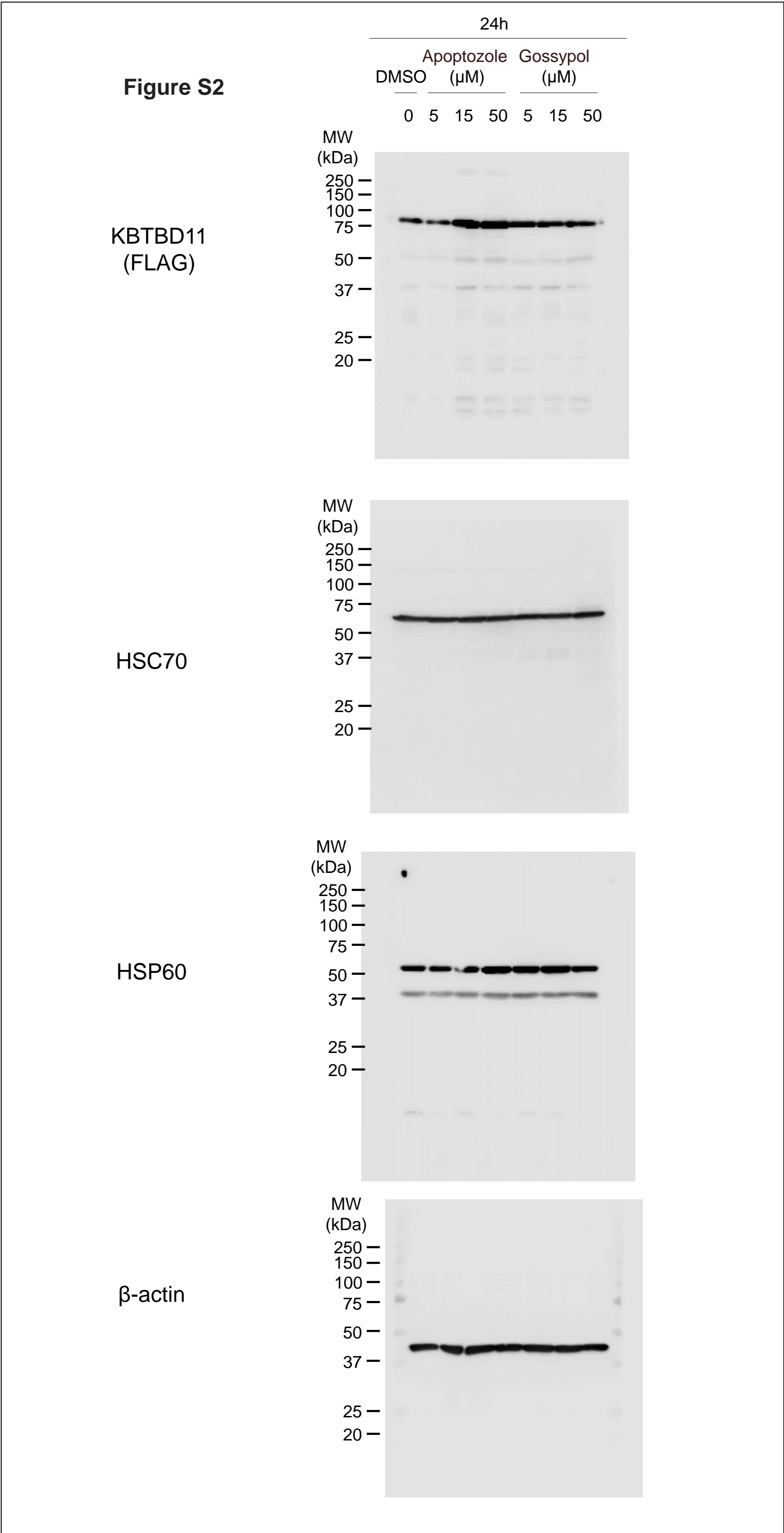

Figure S9

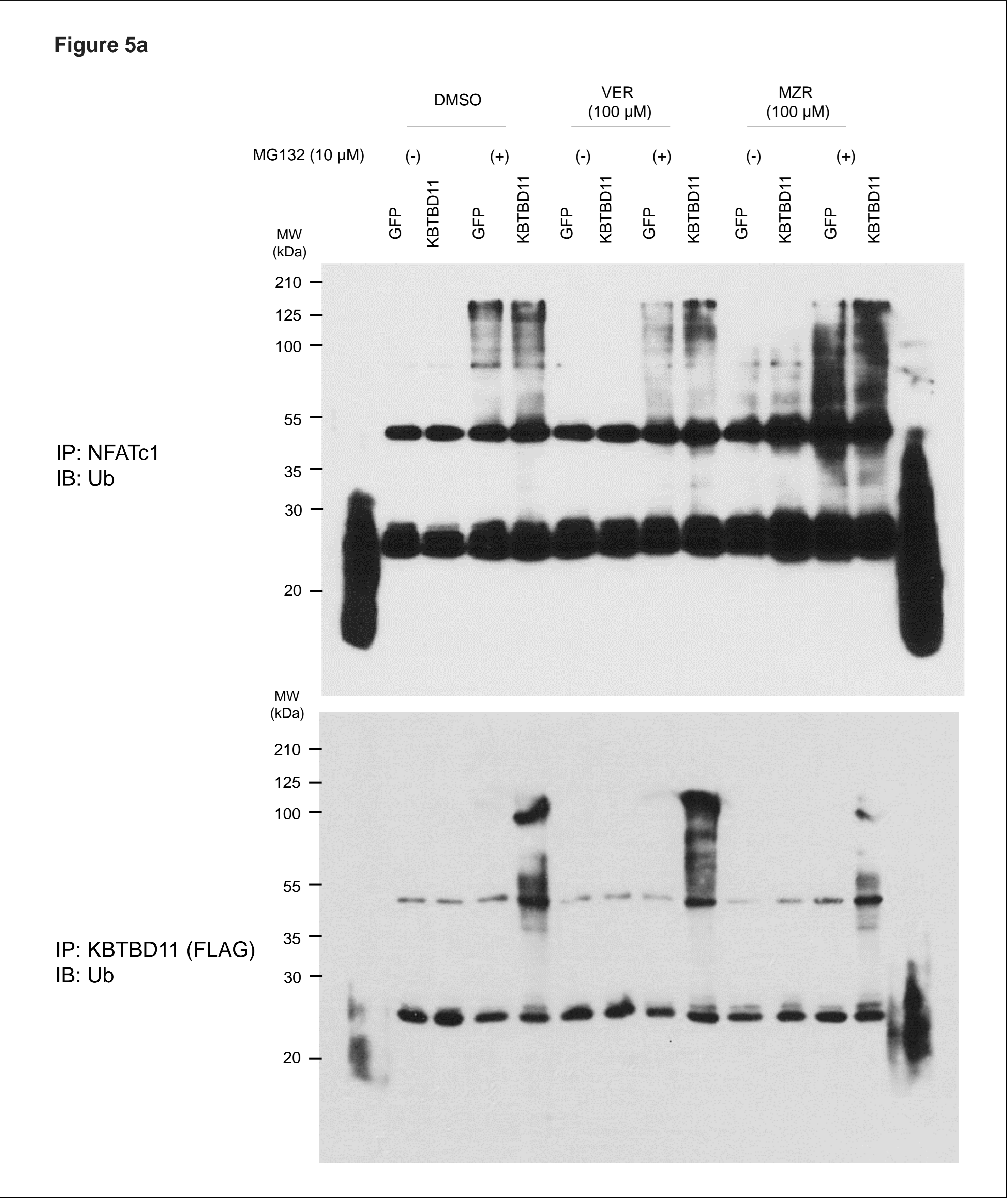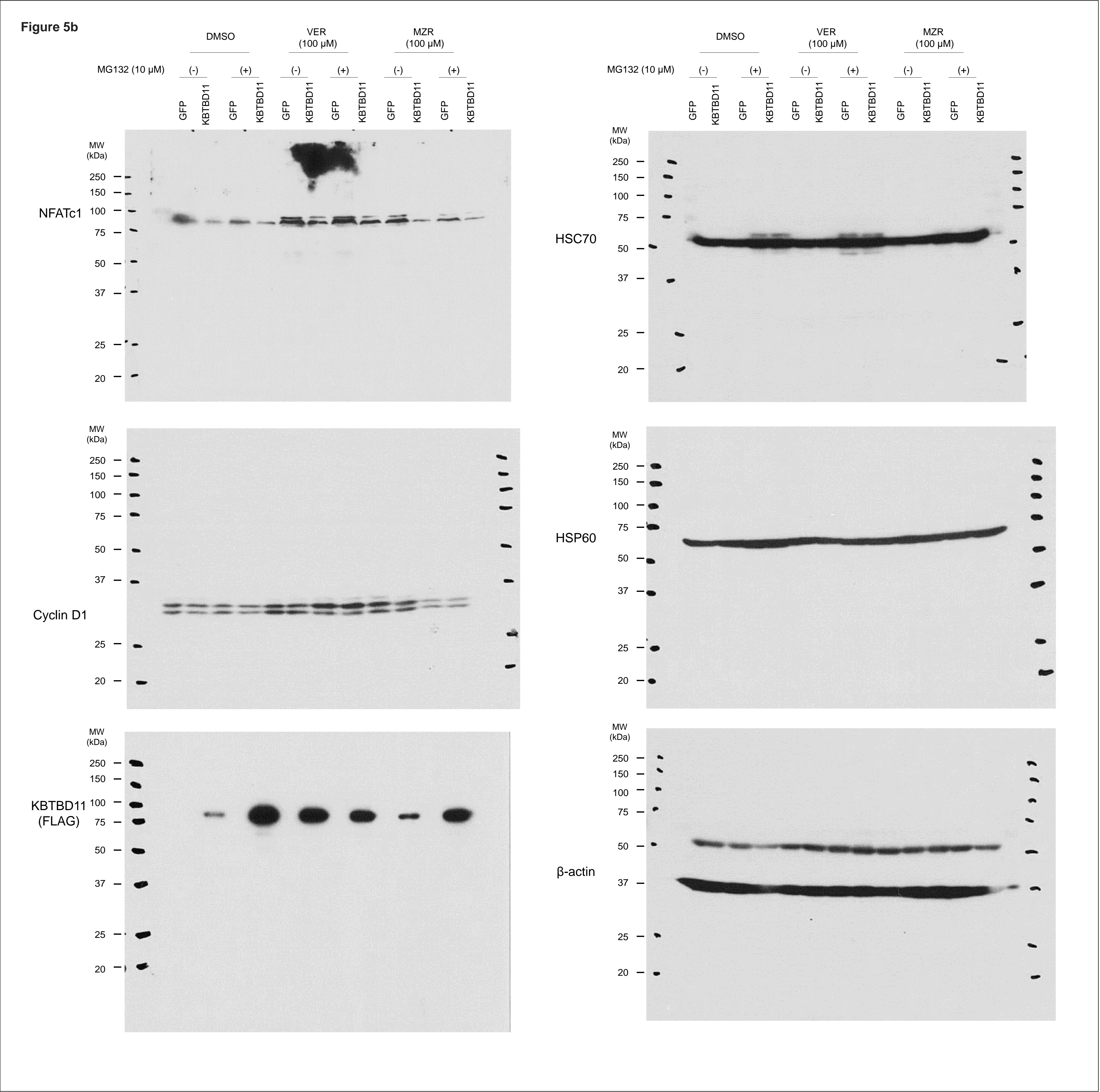

Supplement: Supplementary file 1 — Supplementary Figures. [file 41598_2022_24929_MOESM1_ESM.pdf]
