# Supplementary material for: KBTBD11, encoding a novel PPARγ target gene, is involved in NFATc1 proteolysis by interacting with HSC70 and HSP60
Source: Sci Rep. 2022 Nov 24;12:20273. doi: 10.1038/s41598-022-24929-5 (PMC9700792; doi:10.1038/s41598-022-24929-5)
Supplement: Supplementary file 2 — Supplementary Legends. [file 41598_2022_24929_MOESM2_ESM.docx]

**Supplementary Information**

**KBTBD11, encoding a novel PPARγ target gene, is involved in NFATc1 proteolysis by interacting with HSC70 and HSP60**

Kazuhisa Watanabe^*^, Ayumi Matsumoto, Hidetoshi Tsuda, and Sadahiko Iwamoto

Division of Human Genetics, Center for Molecular Medicine, Jichi Medical University, 3311-1 Yakushiji, Shimotsuke, Tochigi 329-0498 Japan

^*^ Corresponding author: Kazuhisa Watanabe, PhD

Division of Human Genetics,

Center for Molecular Medicine, Jichi Medical University, 3311-1 Yakushiji, Shimotsuke, Tochigi 329-0498, Japan

Phone No: +81-285-58-7341

Fax No: +81-285-44-4902

Email Address: [kwatanabe@jichi.ac.jp](mailto:kwatanabe@jichi.ac.jp)

**Supplementary Table 1**

Forward and reverse primers and EMSA probes.

**Supplementary Figures legends**

**Figure S1: Cullin3 interacts with KBTBD11, HSC70 and HSP60**

3T3-L1 cells were infected either with GFP or FLAG-His-tagged Kbtbd11 adenovirus. After the infection, whole-cell lysates were prepared and immunoprecipitated with the anti-Cullin3 antibody, followed by immunoblotting with anti-FLAG, anti-HSC70, anti-HSP60, and anti-Cullin3 antibodies.

**Figure S2:** **HSC70 and HSP60 affect KBTBD11 protein levels**

(a) 3T3-L1 cells were infected with FLAG-His-tagged Kbtbd11 adenovirus. After 48 h of infection, incubated for 24 h with or without various Apoptozole and Gossypol concentrations, whole-cell lysates were prepared and subjected to western blot analysis using anti-FLAG, anti-HSC70, anti-HSP60, and β-actin antibodies. (b) Quantification of the relative KBTBD11, HSC70, and HSP60 protein levels detected by western blot. The data were normalized to β-actin. n = 3 per group, *p < 0.05, **p < 0.01 vs. DMSO control.

**Figure S3:** Original images for EMSA and ChIP assay in Figure 1.

**Figure S4:** Original images for western blot and silver stain in Figure 2.

**Figure S5:** Original images for western blot in Figure 3.

**Figure S6:** Original images for western blot in Figure S1.

**Figure S7:** Original images for western blot in Figure 4.

**Figure S8:** Original images for western blot in Figure S2.

**Figure S9:** Original images for western blot in Figure 5.
